# Susceptibility to ciprofloxacin of *Escherichia coli* strains isolated from patients with chronic kidney disease

**DOI:** 10.1186/1471-2334-14-S7-P67

**Published:** 2014-10-15

**Authors:** Oana Mariana Ionete, Carmen Avrămescu, Maria Bălăşoiu, Florin Dan Popescu, Lucica Rosu, Ovidiu Zlatian

**Affiliations:** 1 University of Medicine and Pharmacy Craiova, Romania; 2 Carol Davila University of Medicine and Pharmacy, Bucharest, Romania

## Background

Urinary tract infection (UTI) is frequently found in patients with renal failure and is associated with azotemia, reduced urinary flow and impaired urinary concentration.

## Methods

This trial was conducted in the Nephrology Clinic, Emergency County Hospital Craiova, during a period of one year. It included uropathogenic *Escherichia coli* strains isolated from patients with chronic kidney disease. We followed the distribution of these strains according to the stage of kidney disease, age and sex of patients. Also we have tested the susceptibility to ciprofloxacin of these strains.

## Results

According to the glomerular filtration rate and the stage of kidney disease, *Escherichia coli* was associated predominantly with stage five of chronic kidney disease (43.59% women and 12.82% men, with a total of 56.41%), which represents kidney failure (dialysis or kidney transplant required). Proportion of *Escherichia coli* urinary tract infection was significantly higher in patients over 65 years (41.03% women, 5.12% men and a total of 46.15% in this age group). Also the percentage of *Escherichia coli* strains collected from women (84.62%) was higher than men (15.38%). The results regarding the susceptibility to ciprofloxacin revealed the following values: 51.28% susceptibility (20 strains), 7.69% intermediate susceptibility (3 strains) and 41.03% resistance (16 strains). The resistance was associated predominantly with stage five of chronic kidney disease (23.08%, 9 strains respectively).

## Conclusion

*Escherichia coli* urinary tract infections in patients with chronic kidney disease are prevalent in women and in patients over 65 years. *Escherichia coli* strains isolated from patients with chronic kidney disease have a significant level of resistance to ciprofloxacin (41.05%). The severity of *Escherichia coli* urinary tract infection and its poor outcome, with frequent recurrences, are influenced by multiple factors related to sex, age, co-morbidities, multiple admissions and selected resistant strains to antibiotics.

